# Biosynthetic regulatory network of flavonoid metabolites in stems and leaves of *Salvia miltiorrhiza*

**DOI:** 10.1038/s41598-022-21517-5

**Published:** 2022-10-28

**Authors:** Hanting Yang, Hongyan Li, Qian Li

**Affiliations:** State Key Laboratory of Aridland Crop Science, College of Agronomy, Gansu Agricultural University, Lanzhou, 730070 China

**Keywords:** Molecular biology, Plant sciences

## Abstract

Flavonoid secondary metabolites can treat and prevent many diseases, but systematic studies on regulation of the biosynthesis of such metabolites in aboveground parts of *Salvia miltiorrhiza* are lacking. In this study, metabonomic and transcriptomic analyses of different *S. miltiorrhiza* phenotypes were conducted to explore pathways of synthesis, catalysis, accumulation, and transport of the main flavonoid secondary metabolites regulating pigment accumulation. Tissue localization and quantitative analysis of flavonoid secondary metabolites were conducted by laser scanning confocal microscopy (LSCM). A total 3090 differentially expressed genes were obtained from 114,431 full-length unigenes in purple and green phenotypes, and 108 functional genes were involved in flavonoid biosynthesis. Five key phenylpropane structural genes (*PAL*, *4CL*, *ANS*, *3AT*, *HCT*) were highly differentially expressed, and four transcription factor genes (*MYB*, *WRKY*, *bHLH*, *bZiP*) were identified. In addition, six GST genes, nine ABC transporters, 22 MATE genes, and three SNARE genes were detected with key roles in flavonoid transport. According to LSCM, flavonoids were mainly distributed in epidermis, cortex, and collenchyma. Thus, comprehensive and systematic analyses were used to determine biosynthesis, accumulation, and transport of flavonoids in stems and leaves of different *S. miltiorrhiza* phenotypes. The findings will provide a reference for flavonoid production and cultivar selection.

## Introduction

*Salvia miltiorrhiza* Bunge is an important herb in Chinese medicine that was first recorded in Shennong’s herbal classics and is currently widely used to prevent and treat cardiovascular and cerebrovascular diseases^1,2^. Tanshinones and phenolic acids are two major groups with significant pharmacological constituents obtained from *S. miltiorrhiza*, which have important roles in traditional Chinese clinical practice and Western medicine^3,4^. Flavonoids are another active ingredient that are widespread in aboveground parts of *S. miltiorrhiza*^5–7^. Because aerial parts of *S. miltiorrhiza*, are nonmedicinal, aboveground parts are always discarded during harvesting, resulting in huge waste of resources and increased environmental pressure. However, owing to the high flavonoid content and associated antioxidant characteristics, aerial parts of *S. miltiorrhiza* can be developed into natural antioxidants and used in foods.

Flavonoids are a group of bioactive compounds found extensively in foodstuffs of plant origin, and regular consumption of flavonoids is associated with reduced risk of several chronic diseases^9^. Flavonoids are important secondary metabolites involved in plant growth and development but have also been studied in many plants because of excellent antioxidant and anticancer effects^5,7^. Flavonoids primarily include anthocyanins (red to purple pigments), flavonols (colorless to light yellow pigments), flavanols (colorless pigments that turn brown after oxidation), and procyanidins (PAS)^8^. In addition, flavonoid metabolite distribution and accumulation vary with plant species, tissues, organs, development stages, and growth conditions^10,11^.

The growing evidence of the versatile health benefits of flavonoids including anti-inflammatory, antioxidant, antiproliferative and anticancer activity^8,11^. Diets that include flavonoid containing products are currently recommended^12^, further studies are necessary for confirmation of the beneficial effects, such as determine their mechanisms of action at the molecular level, to lay a foundation for the rational promotion of diet rich in flavonoids. Biosynthesis of flavonoids shares the same upstream pathway as lignin and phenolic acid biosynthesis, which is derived from the phenylpropane pathway^13^. In addition, biosynthesis and accumulation of flavonoids in *S. miltiorrhiza* are influenced by a series of structural genes, including *PAL, C4H, 4CL, CHS, CHI, FLS, FNS, DFR, F3H, F3′H, F3′5′H, ANS, 3GT,* and *3AT*, and regulatory transcription factors, including *MYB, bHLH, bZiP, WD40*, and *WRKY*, or transcriptional complexes (*MYB-bHLH-WD*) in the flavonoid metabolic pathway. Genes (e.g., *HCT*) from other pathways can also be involved. Although expression of those genes affects the formation of color in plant organs^14,15^, biological and abiotic factors, especially hormones and environmental factors, also regulate flavonoid biosynthesis^16^.

Color phenotypes of plants organs are associated with the biosynthesis and accumulation of flavonoids^8,17,18^. For example, a decrease in flavonoids/anthocyanins was the primary reason leaf color changed in purple-leaf tea plant^19,20^. In addition, the purple inner leaves of purple ornamental cabbage result from a high level of anthocyanin biosynthesis, a high level of chlorophyll degradation, and an extremely low level of chlorophyll biosynthesis^18^. In *Suaeda salsa*, two anthocyanins are responsible for red in leaves^19^, and in *Ziziphus jujuba*, flavonoids are the major differential metabolites determining leaf color^20^.

To better understand the mechanism of color change and the dynamic changes in related metabolites and genes expression levels, metabolic and transcriptional profiles of stems and leaves of *S. miltiorrhiza* purple and green phenotypes were investigated in this work. Differentially accumulated metabolites (DAMs), including flavonoids and phenolic acids, and differentially expressed genes (DEGs) in flavonoid biosynthesis pathways were determined. To further explore the biosynthesis, accumulation, and transport of flavonoids in aboveground tissues of *S. miltiorrhiza*, laser scanning confocal microscopy (LSCM) with 2-Aminoethyl diphenylborinate (NA) taining-induced fluorescence was performed. On the basis of the analyses, the biosynthesis, accumulation, and transport of flavonoids in stems and leaves of different phenotypes were determined. The work revealed dynamic changes in metabolites and molecular mechanisms regulating underlying color changes in *S. miltiorrhiza* stem and leaf.

## Results

### Comparison of morphological phenotypes

**Figure 1:**
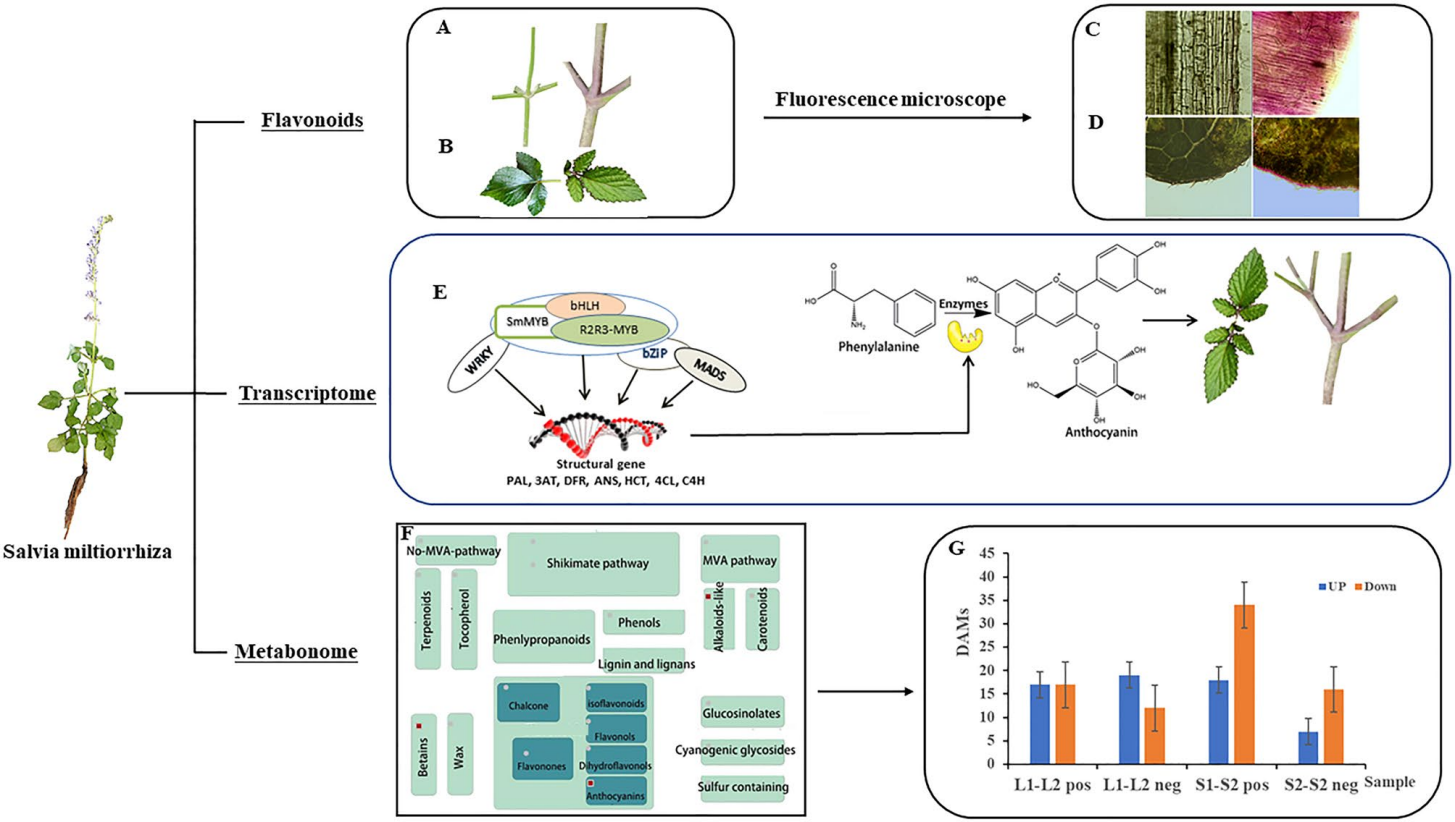
Response of *S. miltiorrhiza* Phenotypes to flavonoids. **(A)** Surface view of green and purple stem internode. **(B)** Surface view of green leaf and the leaves with purple edges. **(C)** Periderm tissue of green and purple stems under fluorescence microscope. **(D)** Periderm and mesophyll tissue of green leaves and the leaves with purple edges under fluorescence microscope. **(E)** A model to explain the internal mechanism of purple pigmentation in stems and leaves of *S. miltiorrhiza*. **(F)** The main secondary metabolic pathway in *S. miltiorrhiza*. **(G)** DAMs in different phenotypes of *S. miltiorrhiza* stems and leaves (L1: green leaf, L2: the leaves with purple edges; S1: green stem, S2: purple stem, pos indicated positive ion mode; neg indicated negative ion mode).

Morphological phenotypes of green and purple stems in *S. miltiorrhiza* were significantly different. Purple plants had a better agronomic phenotype than that of green plants, with tall plants, thick stems, and purple stem skin with more villi. In addition, the color of buds and leaves on purple plants was different from that of old leaves (Fig. 1A). The edges of young buds and leaves were purple, and the edge of old leaves was also purple. In the comparison of leaves with purple edges and completely green leaves, leaves with purple edges were yellowish green, sharply serrated edges were obvious, and some of the stems were purple. In addition, there was more fluff on the back (Fig. 1B). Microscopic observation indicated the pigment of purple stems primarily accumulated in several layers of cells below the epidermis (Fig. 1C). Therefore, stem skin tissues were observed by hand section, and edge tissues of green and purple leaves were compared. Purple pigment was primarily in the epidermal cells at leaf edges (Fig. 1D). Thus, the purple and green aboveground parts were taken as the materials, we analyzed them using metabonoics (Fig. 1F) and transcriptomics (Fig. 1E), and differentially expressed genes and metabolites were identified (Fig. 1G). In order to establish a dynamic accumulation model of biosynthesis pathways of *S. miltiorrhiza* using transcriptome and metabolome, and the biological transport pathway leading to accumulation and transport of purple pigment was investigated (Fig. 1).

### Genome sequencing, assembly, and annotation

A total of 76.95 GB of data were measured using a DNBSEQ sequencing platform to sequence the test materials (S1, S2, L1, L2). The clean data of each sample reached 6.3 GB; the Q20 value of obtained sequences was greater than 96%; the percentage of Q30 bases was ≥ 90%; and the GC content was more than 43% (Supplementary Table S2). A total of 40,077 simple sequence repeats (SSR) loci were obtained. The 19,216 unigene sequences included 93 repeat units, of which dinucleotide repeat was the dominant repeat type (55.27%), followed by mononucleotide repeat (15.5%) and trinucleotide repeat (23.1%). The dominant units of mononucleotide and dinucleotide repeats were A/T and AG/TC, respectively (Table 1). In the comparison with the reference database, 114,431 unigenes were successfully annotated, and 92,326 (80.68%) genes were annotated in the Non-Redundant Protein Sequence Database (NR), of which 60,704 (65.75%) genes were consistent with the characteristics of *Salvia splendens* and 3566 genes were unique to *S. miltiorrhiza*. The results indicated that the quality of sequencing data was high, and therefore, the data met the requirements of transcriptome analysis.

**Table 1:** Transcriptome SSR repeat type and number.

Repeats motifs	Duplicate times								Total	Ratio (%)
	4	5	6	7	8	9	10	> 10		
Mono-nucleotide	–	–	–	–	–	–	1445	6198	6198	15.5
Di-nucleotide	–	–	6791	4410	3540	2261	1468	3679	22,149	55.27
Tri-nucleotide	–	4980	2171	1013	607	141	93	214	9219	23.1
Quad-nucleotide	–	205	53	8	5	12	3	6	292	0.73
Penta-nucleotide	558	172	30	8	7	2	6	1	784	1.96
Hexa-nucleotide	1088	196	95	39	10	3	2	2	1435	3.58
Total	1676	5553	9140	5478	4169	2419	1572	10,100	40,077	–
Ratio (%)	4.18	13.9	22.8	13.7	10.4	6.04	3.92	25.2	–	–

**Figure 2:**
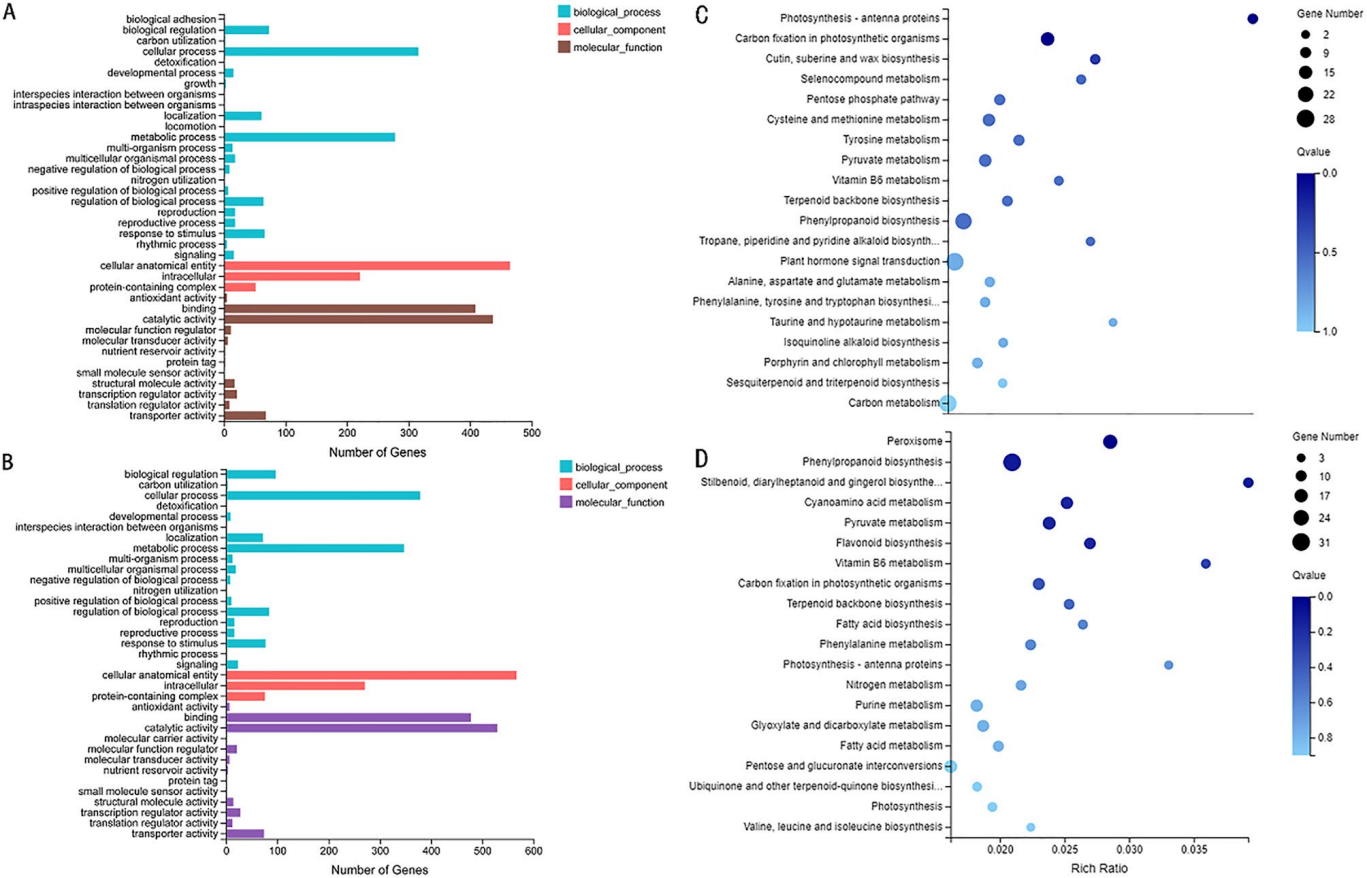
**(A)** GO functional classification of differentially expressed genes in L1 (green leaf), and L2 (the leaves with purple edges) in *S. miltiorrhiza*. **(B)** GO functional classification of differentially expressed genes in S1 (green stem), and S2 (purple stem). **(C)** Scatter plots of the KEGG pathway enrichment statistics in L1–L2. **(D)** Scatter plots of the KEGG pathway enrichment statistics in S1–S2.

According to the criteria *q*-value ≤ 0.05 and log2 (fold-change) ≥ 1, 1299 DEGs were identified in S1–S2, including 666 up-regulated genes and 633 down-regulated genes, and 1091 DEGs were identified in L1–L2, including 393 up-regulated genes and 698 down-regulated genes (Fig. S1). Gene Ontology analysis (GO) and Kyoto Encyclopedia of Genes and Genomes (KEGG) pathway enrichment analyses were performed on DEGs. In the GO analysis of the S1–S2 group, 481, 572, and 805 DEGs were assigned to the categories biological processes, molecular functions, and cellular components, respectively, with DEGs further classified into 35 functional subcategories (Fig. 2A). In the L1–L2 group, 402, 469, and 676 DEGs were assigned to the categories biological processes, cellular components, and molecular functions, respectively, with DEGs further classified into 38 functional subcategories (Fig. 2B).

To understand biological functions and gene interactions, DEGs were annotated by the KEGG database (www.kegg.jp/kegg/kegg1.html) ^21^. In the S1–S2 group (Fig. 2C), 473 of 1299 DEGs were in 122 KEGG pathways, including cellular processes, environmental information processing, genetic information processing, metabolism, and organismal systems. In the L1–L2 group (Fig. 2D), 438 of 1091 DEGs were in 113 KEGG pathways. In the S1-S2 group, the main enriched metabolic process was phenylpropanoid biosynthesis, and twelve DEGs were involved in flavonoid biosynthesis. In further analysis of the DEGs, six Glutathione S-transferase (GST) genes, nine ABC transporter genes, 22 MATE genes, and three SNARE (soluble NSF attachment protein receptor) genes were also detected, which may have important roles in transporting anthocyanin to plant vacuoles^7^. In the L1–L2 group, the main enriched metabolic process was phenylpropanoid biosynthesis and carbon metabolism. Further analysis revealed eight GST genes and seven ABC transporters.

**Figure 3:**
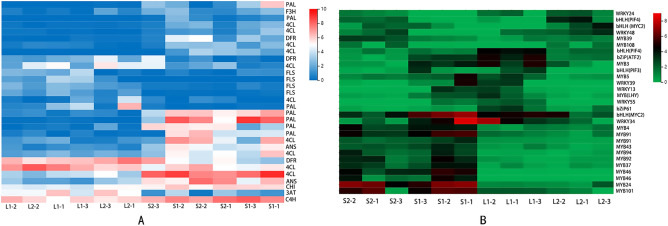
**(A)** Expression patterns of flavonoids biosynthetic genes in stems and leaves samples of *S. miltiorrhiza*. **(B)** Expression of flavonoids biosynthetic transcription factors in nine samples of *S. miltiorrhiza*. The horizontal axis represents log2 (FPKM + 1) of the sample and the vertical axis represents genes. L1(green leaf), L2(the leaves with purple edges), S1(green stem), S2 (purple stem).

Anthocyanins and flavonoid secondary metabolites are the main components that affect the color of petals, stems, leaves, and fruits^6,7^. In the enrichment analysis, *PAL, C4H/CYP73A, 4CL, DFR, ANS, FLS, CHI, F3H*, and *3AT* were significantly different in the flavonoid biosynthesis pathway. Hierarchical Cluster Analysis (HCA) was performed on DEGs with a pheatmap package (Fig. 3A, Supplementary Table S3).

Flavonoid contents in plants are strictly regulated by complex regulatory networks. Transcription factors (TFs), including members of *MYB, bHLH, MADS, WRKY, bZiP*, and *WD40* families, have important roles in regulating plant flavonoid biosynthesis ^22^. In this study, differential expression levels of *MYB, bHLH, bZiP*, and *WRKY* were detected. It is worth mentioning that the *MYB* family had the largest number and the greatest differences among all TF genes detected. The differential expression of TF genes was most obvious in stem tissue (Supplementary Table S4, Fig. 3B). Transcription factors *MYC2* and *WRKY34* were expressed at higher levels in green stems than in purple stems. The findings indicated that the TFs were closely associated with phenotypic differences in color in different tissues.

### Metabolomic analysis

A representative sample (S1, S2, L1, L2) was selected from each group (three biological replicates) of samples to display the base peak ion Base Peak Chromatogram (BPC) chromatogram. The total ion flow chromatogram of different phenotypes of stems and leaves of *S. miltiorrhiza* is shown in Fig. S2. In the BPC diagram, the peak results of the tested samples were good, the peak shape was good, and the peak area was large. However, there were some differences among the overall contours of the four samples. To reveal the component differences between the different phenotypes of *S. miltiorrhiza*, metabolite separation and detection were conducted. After data preprocessing, in leaves, 10,636 metabolites were identified in positive ion mode, and 5231 metabolites were identified in negative ion mode. In stems, 6264 metabolites were identified in positive ion mode, and 3503 metabolites were identified in negative ion mode (Table 2).

**Table 2:** Statistical table of compound detection.

	Mode	Number of metabolites	Number of metabolites with identification information
Stem	Pos	6264	816
	Neg	3503	466
Leaf	Pos	10,636	944
	Neg	5231	524

**Figure 4:**
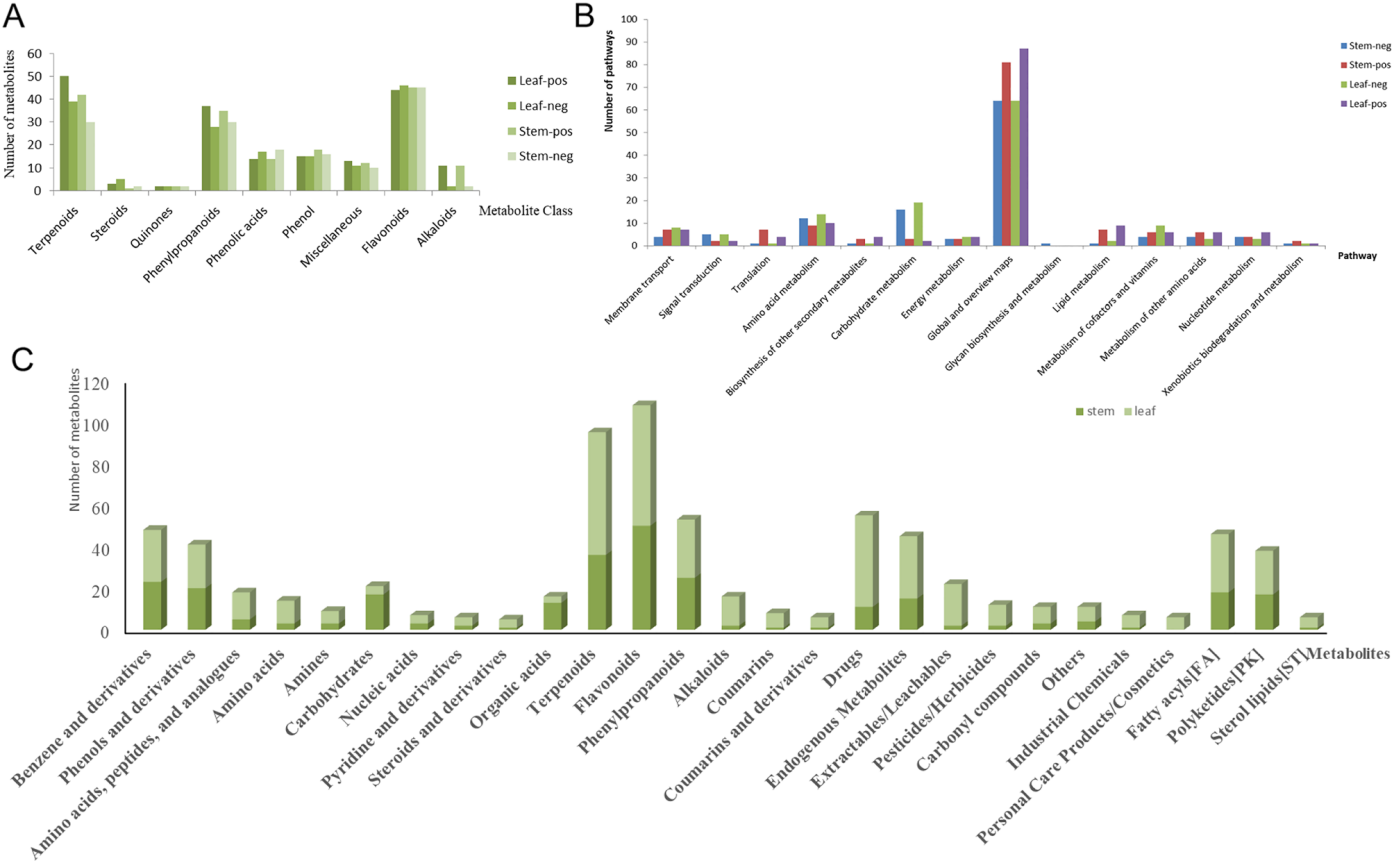
**(A)** Metabolite classification bar chart in BGI library. **(B)** Bar chart of metabolite KEGG function annotation. **(C)** Bar chart of metabolite classification.

Metabolites identified in *S. miltiorrhiza* leaves were classified in the Beijing Genomics Institute (BGI) library. Of the metabolites, 44 were flavonoids, 50 were terpenoids, 37 were phenylpropanoids, 14 were phenolic acids, 15 were phenols, 13 were classified as miscellaneous, and 3 were steroids. Eleven metabolites were alkaloids (alkaloids and glycosides), and two were quinones (quinones and glycosides). Metabolites identified in *S. miltiorrhiza* stems were also classified in the BGI library. Of the metabolites, 45 were flavonoids and glycosides, 42 were terpenoids and glycosides, 35 were phenylpropanoids and glycosides, 18 were phenolic acids and glycosides, 14 were phenols and glycosides, 12 were in other classes, and one was in the steroids and glycosides group. Eleven metabolites were alkaloids and glycosides, and two metabolites were quinones and glycosides. Thus, flavonoids and glycosides, terpenoids and glycosides, and phenylpropanoids and glycosides were the main metabolites identified in all samples of aboveground parts of *S. miltiorrhiza* (Fig. 4A).

Metabolites were classified and annotated in Human Metabolome Database (HMDB and KEGG databases^23^. All metabolites were classified in three main pathways in the KEGG database, which were environmental information processing and metabolism and genetic information processing. In leaf tissue, environmental information processing had two branches: membrane transport (77.78%) and signal transduction (22.22%). Genetic information processing also had two branches: translation (80%) and folding, sorting, and degradation (20%). All metabolites accounted for the largest proportion (65.93%) in global and overview maps. In stem tissue of *S. miltiorrhiza*, two branches under environmental information processing included membrane transport (77.78%) and signal transduction (22.22%). Under genetic information processing, there was a branch of translation. All metabolites accounted for the largest proportion (65.32%) in global and overview maps (Fig. 4B). In leaves, all metabolites were divided into four categories: compounds with biological roles (31.42%), phytochemical compounds (41.09%), others (11.18%), and lipids (16.31%). Flavonoids and terpenoids accounted for large proportions of phytochemical compounds, with 37.5% and 36.03%, respectively. In stems, terpenoids (32.8%), flavonoids (32.22%), and phenylpropanoids (15.56%) were the three most abundant phytochemical compounds (Fig. 4C).

**Figure 5:**
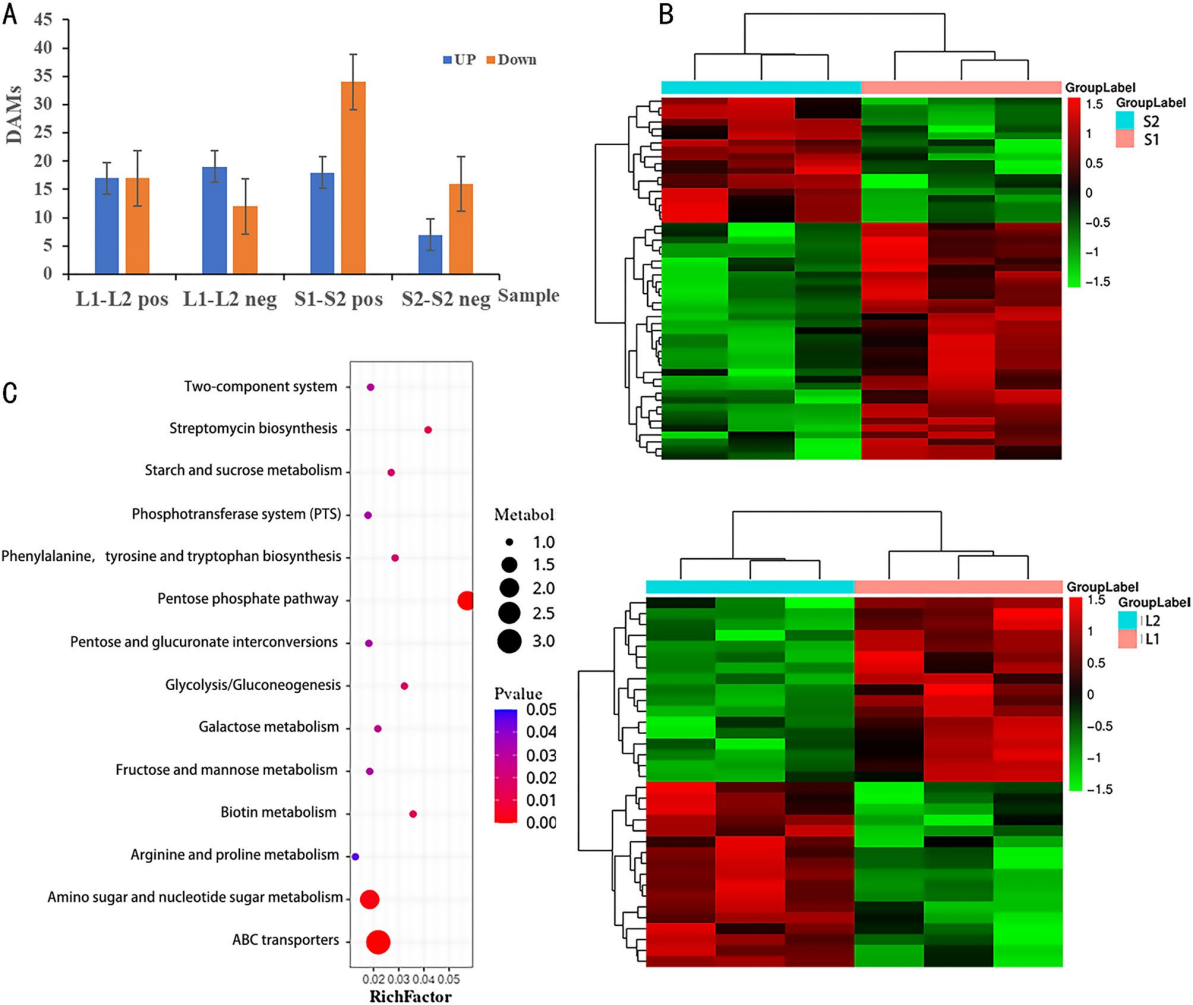
**(A)** Statistical map of differential metabolites (DAMs). **(B)** The Hierarchical cluster analysis of differential accumulated metabolites (DAMs) in L1–L2, S1–S2 of *S. miltiorrhiza*. **(C)** Bubble Diagram of metabolic pathway enrichment analysis in L1–L2.

The DAMs between green and purple cultivars were determined according to the criteria of fold-change ≥ 1.2 or ≤ 0.83 and P-value < 0.05. In green stems and purple stems, 23 DAMs were screened, of which seven were up-regulated and 16 were down-regulated (Supplementary Table S5). In green leaves and leaves with purple edges, 34 DAMs were screened, of which 17 were up-regulated and 17 were down-regulated (Supplementary Table S6, Fig. 5A, Fig. S3). Then, an HCA was performed on the accumulation pattern of metabolites among different samples^24^. The HCA revealed two clusters of metabolites, with one the metabolites before the color change (S1, L1) and the other the metabolites after the color change (S2, L2) (Fig. 5B).

To better identify which pathways are enriched between different DAMs, the metabolic pathways of all DAMs were enriched and analyzed based on the KEGG database. Forty-eight metabolites were enriched in 46 metabolic pathways, and 36 metabolites were enriched in the biosynthesis of secondary metabolites. The pathways with significant enrichment (*P* < 0.05) were selected. In stems, seven DAMs were annotated in the metabolic pathway (guanosine, aflatoxin G2, estriol, arachidonic acid, 5'-S-methyl-5ʹ-thioadenosine, 4-coumaric acid, and sinapic acid), and four DAMs were annotated in the secondary metabolite biosynthesis pathway (coumarin, 4-coumaric acid, sinapic acid, and phlorizin) (Table 3). For leaves, bubble plots show significant enrichment of DAMS (*P* < 0.05) in 14 pathways. The largest proportion of DAMs was annotated to the pentose phosphate pathway. The largest number of DAMs was annotated to ABC transporters, accounting for 17.65%. The metabolites (d-(+)-glucose, biotin, d-(+)-xylose), d-(+)-glucose, and d-(+)-xylose were involved in the pentose phosphate pathway and antibiotic biosynthesis. The differential metabolite d-(−)-quinic acid was involved in the metabolic pathway of phenylalanine, tyrosine and tryptophan biosynthesis, and d-(+)-glucose was involved in almost all other metabolic pathways (Fig. 5C).

**Table 3:** Metabolic pathway enrichment with Pvalue < 0.05 in S1–S2.

Pathway	Count	P value	DAMs names
Metabolic pathways	7	0.0018	Guanosine, aflatoxin G2, estriol, arachidonic acid, 5ʹ-S-methyl-5ʹ-thioadenosine, 4-coumaric acid, sinapic acid
Biosynthesis of secondary metabolites	4	0.0113	Coumarin, 4-coumaric acid, sinapic acid, phlorizin
Cysteine and methionine metabolism	1	0.0463	5ʹ-S-methyl-5ʹ-thioadenosine
Arachidonic acid metabolism	1	0.0549	Arachidonic acid
Purine metabolism	1	0.069	Guanosine
ABC transporters	1	0.098	Guanosine
Degradation of aromatic compounds	1	0.1804	4-Coumaric acid
Biosynthesis of antibiotics	1	0.5483	Aflatoxin G2

### Network of differentially accumulated metabolites regulated by differentially expressed genes

To further correlate gene expression patterns with metabolite accumulation, coexpression analysis was applied to metabolome and transcriptome data from *S. miltiorrhiza* purple and green stems and leaves. Expression levels of *PAL, 3AT, 4CL, ANS, HCT, MYB43, MYB101, MYB108, MYB94, WRKY24*, and *WRKY48* were higher in purple samples, whereas expression levels of *DFR* and *CYP73A* were higher in green samples (Supplementary Tables S3, S4). It was hypothesized that those key genes and transcription factors were involved in the regulation of flavonoid and phenolic acid biosynthesis, thereby affecting the color of *S. miltiorrhiza* stem and leaf. The anthocyanin accumulation in purple characters was closely related to the high expression of the synthases involved in anthocyanin biology.

**Figure 6:**
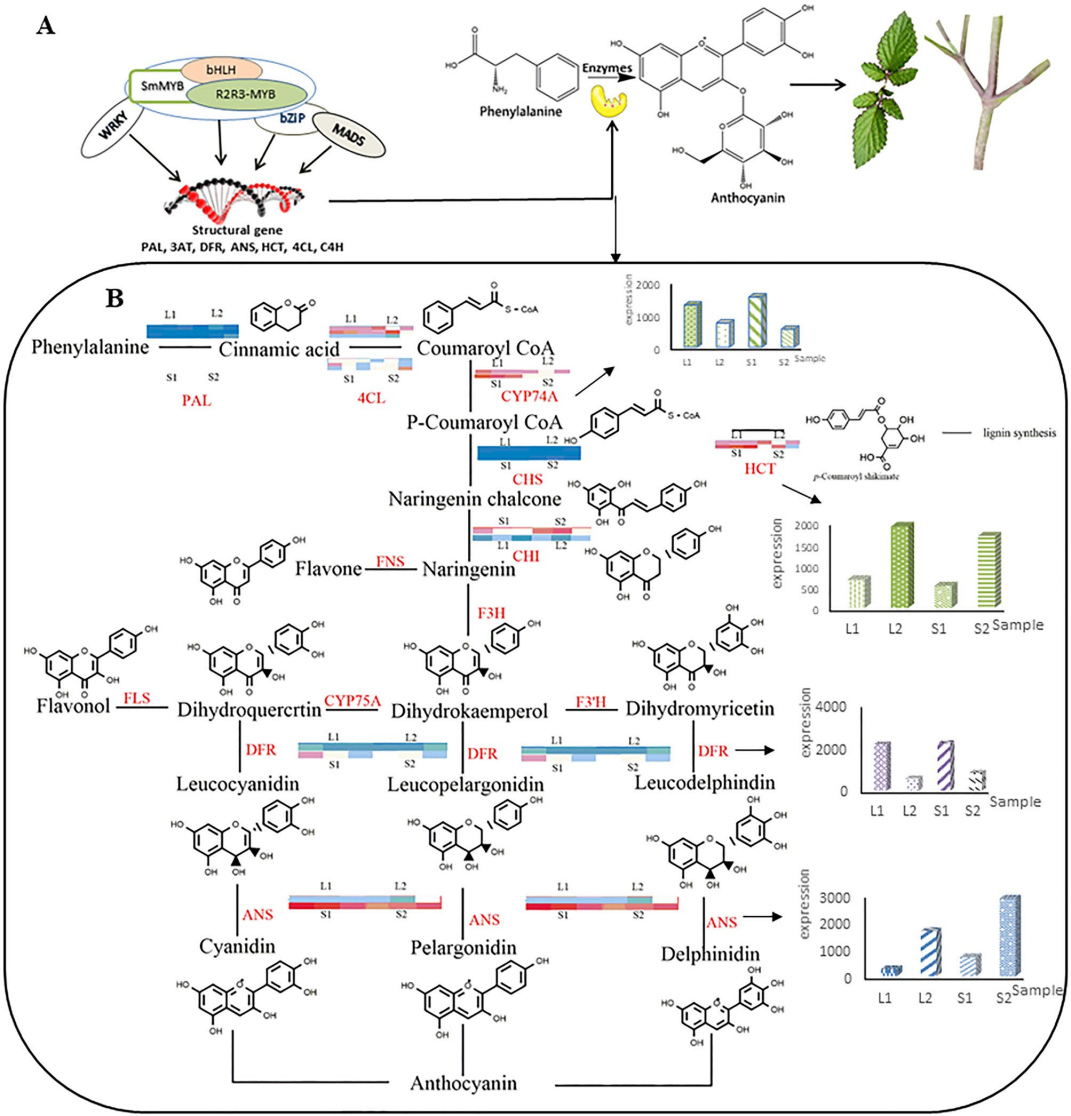
Molecular regulation of color phenotype characters of *S. miltiorrhiza*. **(A)** A working model to explain the internal mechanism of purple pigmentation in the aboveground part of *S. miltiorrhiza*. **(B)** Schematic diagram of flavonoids biosynthesis and regulation in *S. miltiorrhiza*. The redder the color of the color block, the higher the expression, and the bluer the color, the lower the expression.

In the main phenylpropanoid and flavone biosynthesis pathway, strong activation and expression of all structural genes eventually led to the production of many anthocyanins. In the flavonoid biosynthesis pathway, under the catalysis of *MYB, bHLH, MADS, WRKY*, and *bZiP* families, yields of flavones and anthocyanins also increased on a large scale. Anthocyanins are a group of water-soluble pigments that help to form various colors of plants. Flavonoids, especially those in the anthocyanin biosynthesis pathway, led to the accumulation of purple pigment (Fig. 6).

### Reverse-transcription quantitative PCR of transcriptomic data

**Figure 7:**
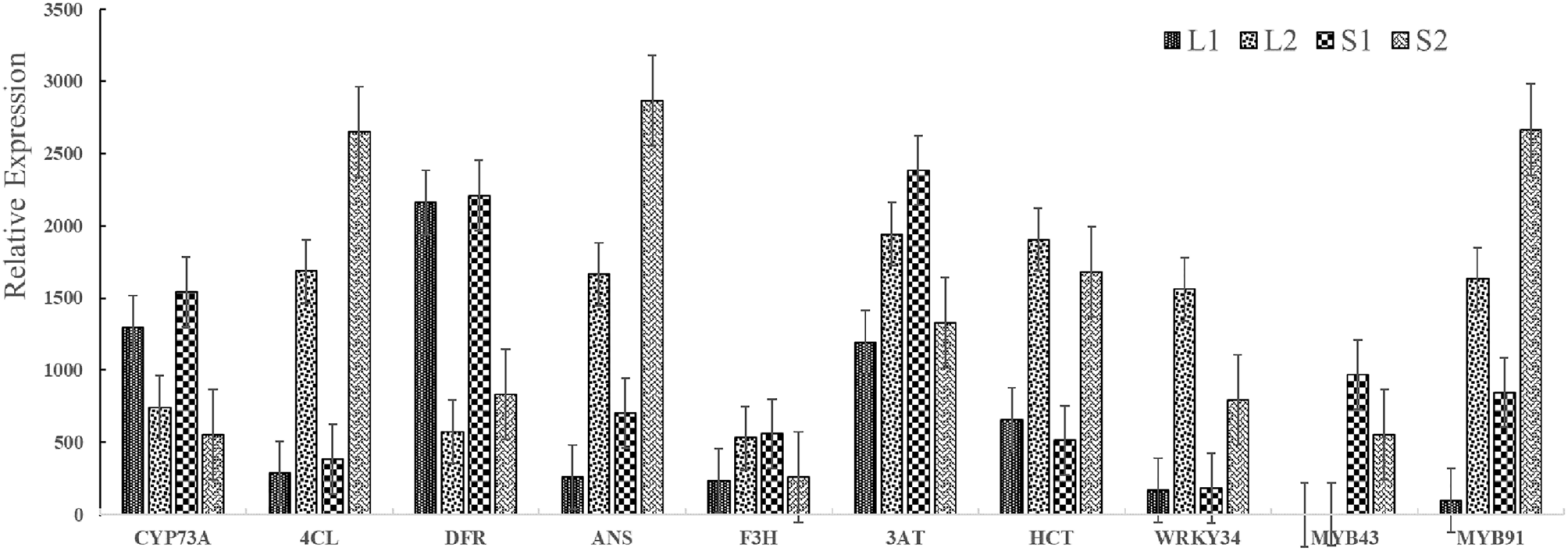
Expression levels of 10 genes involved in the flavonoids biosynthetic pathway by qRT-PCR analysis.

To validate transcriptome information, 10 DEGs were selected to validate the sequencing results. The comparative CT method (2−ΔΔCTmethod) was used to quantify gene expression. Reverse-transcription quantitative PCR (RT-qPCR) indicated that six genes had higher expression levels and four had lower expression levels in the purple phenotype than in the green phenotype (Fig. 7). The RT-qPCR results were consistent with those obtained with RNA-seq.

### Distribution dynamics of flavonoids by laser scanning confocal microscopy

**Figure 8:**
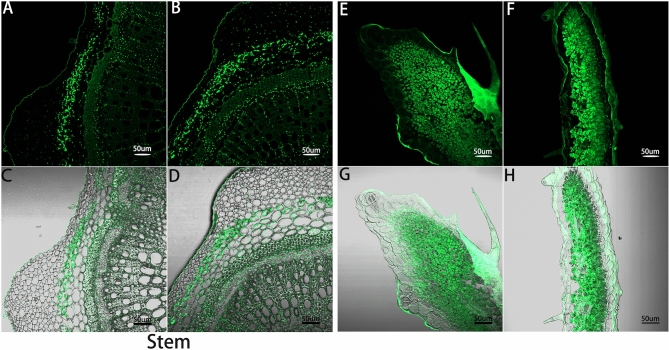
Leaves and stems were stained with NA solution and observed with CLSM (50 µm). The figure showed the distribution of flavonoids in the cross section of *S. miltiorrhiza* after staining. **(A,B,E,F)** indicated the distribution of flavonoids in the AF488 channel. **(C,D,G,H)** Indicated the distribution of flavonoids in superimposed bright field of AF488 channel. **(A,C)** Indicated the purple stem. **(B,D)** Indicated the green stem; **(E,G)** indicated the leaf with purple edge. **(F,H)** Indicated the green leaf.

To investigate locations of flavonoids in purple and green characters from *S. miltiorrhiza*, frozen sections were prepared and observed. At the excitation wavelength of 488 nm, samples showed chloroplast autofluorescence before staining. The interference was eliminated by subtracting the background signal. After staining with NA solution, epidermis, glandular hairs, and thick horn tissue cells of stems in *S. miltiorrhiza* showed strong fluorescence. In addition, a small amount of fluorescence also appeared in the phloem. The observations suggested that flavonoids were biosynthesized and accumulated in epidermis, villi, collenchyma, phloem, and fascicle of *S. miltiorrhiza* (Fig. 8A–D). Flavonoids in *S. miltiorrhiza* leaves were primarily distributed in external tissues such as glandular hair and epidermis (Fig. 8E–H), possibly to prevent microbial invasion and UV radiation damage, and also accumulated in palisade and spongy tissues ^25^. Because methanol is volatile and strongly solubilizes flavonoids, it acts as a solvent of color developing agent. Therefore, flavonoids are dissolved and diffused in the dyeing process, and a small amount of fluorescence can appear in some parts. The content of flavonoids in the green phenotype was significantly higher than that in purple phenotype, which might be because the lower expression level of *ANS* resulted in a decrease in anthocyanin biosynthesis and accumulation in green samples but an increase in flavonoid biosynthesis and accumulation.

## Discussion

The color of plant tissues and organs is determined primarily by the accumulation of flavonoids. Flavonoids, phenolic acids, and lignin are all in downstream branches of the phenylpropane metabolic pathway^20,26^. Expression levels of the main structural genes or TFs in the phenylpropane metabolic pathway can determine final flavonoid biosynthesis and then affect formation of color phenotypes. In this study, five key phenylpropane structural genes (*PAL, 4CL, ANS, 3AT, HCT*) had higher expression in purple than in green samples, and two key anthocyanin structural genes (*DFR, CYP73A/C4H*) had higher expression in green than in purple samples. As key upstream enzyme genes of the phenylpropane pathway, *PAL, 4CL*, and *CYP73A* affect proportions in the metabolic flow of flavonoids, phenolic acids, and lignin^1^. The key enzymes in anthocyanins biosynthesis are *DFR, ANS*, and *3AT*^6^. Overexpression of *SmANS* also increases anthocyanin accumulation and alters phenolic acids content in *S. miltiorrhiza*^17^. The genes *ANS, DFR*, and *F3′5′H* also have key roles in regulating the biosynthesis of anthocyanins in purple stems of *Astragalus membranaceus*^13^. The *3AT* from *Perilla frutescens* can be used as a new molecular tool to modify anthocyanin structure and further increase flower color by recombinant DNA technology^27^. The gene *HCT* mainly controls the metabolic flow between lignin and the flavonoid branches. In *Arabidopsis*, *HCT* can inhibit the synthesis of lignin, which redirects metabolic flux to flavonoids via chalcone synthase activity^28^. In *S. miltiorrhiza* leaves in this study, expression level of *HCT* and content of flavonoids (rhusflavanone, herbacetin, and ipriflavone) were significantly higher in the purple phenotype than in the green phenotype. Therefore, *HCT* might be a positive regulator of flavonoid biosynthesis and thereby affect the color of organs and tissues in *S. miltiorrhiza*.

The structural genes that catalyze the formation of flavonoids in *S. miltiorrhiza*, are regulated by single or multiple TFs as well as complexes formed between TFs^14^. Transcription factors *MYB, AP2/ERFs, WRKY, bHLH, MADS, WD40, zinc finger, NACs, bZiP*, and *LBD* may be involved in the formation of white and purple flowers in *S. miltiorrhiza*^7^. In this work, *MYB, WRKY, bHLH*, and *bZiP* also had important roles in pigment formation of *S. miltiorrhiza* stems and leaves. The regulatory network of *MYB* in *S. miltiorrhiza* is important and complex and therefore can provide useful information to improve the growth and defense ability of *S. miltiorrhiza* as well as the production of active compounds. Transcription factors *MYB58* and *MYB63* can activate *PAL, C4H, 4CL*, and other genes involved in lignin biosynthesis in Arabidopsis^29^. In *S. miltiorrhiza*, *MYB37* is in the same subgroup as *MYB58* and *MYB63*. Therefore, it was inferred that *MYB37* had a similar role in lignin biosynthesis^30^. *Arabidopsis thaliana* *MYB4* can interfere with the transcriptional activity of MBW complexes or inhibit biosynthesis of phenylalanine by repressing expression of the gene encoding arogenate dehydratase 6^31^, which catalyzes the last step in phenylalanine biosynthesis that produces the precursor of flavonoid biosynthesis. In the current study, the green phenotype had high content of *MYB4e*, indicating that the gene had an obvious inhibitory effect on phenylalanine and thereby affected biosynthesis of flavonoids. The color variation in sweet gum young and senescent leaves is attributed to the composition of anthocyanidins produced by transcriptional regulation of *F3′H* and *F3′5′H* by *MYB5, MYB113*, and *MYB123*^32^. Therefore, because the *MYB3* and *MYB5* in *S. miltiorrhiza* are in the same group, the two TFs are also likely involved in anthocyanin biosynthesis^28^. The TFs *MYB5-like* and *bHLH* have roles in the first step of flavonoid production in flowers and immature fruits, and expression patterns of those TFs may be the key to determine differences in flavonoids in pomegranate flowers and fruits^33^. In addition, *MYB37* has an important and positive role in plant response to abscisic acid (ABA) and drought stress^34^. The interaction of TFs *MYB21* and *MYB24* affects the regulation of jasmonic acid^35^, and *SmMYB39* participates in regulation of the rosemary acid pathway by inhibiting the transcription of key enzyme factors^36^.

The *WRKY* family is one of the largest TF families in plants, and it regulates the growth and development of plants^37^. The TF *WRKY55* positively regulates leaf senescence and defense by regulating the transcription of reactive oxygen species (ROS) and salicylic acid (SA) biosynthesis-related genes in Arabidopsis^38^. The TF *WRKY34* interacts with the VQ20 protein to regulate pollen development and function^39^. The *bHLH* family TF *PIF4* is necessary for plants to adapt to light and high ambient temperature, and interaction between *PIF4 and PIF4* interacting proteins establishes a strong network for plants to respond to changes in the environment^40^. In addition, phytochrome interaction factors PIF4 and PIF5 negatively regulate anthocyanin synthesis in Arabidopsis seedlings under red light^41^. In plant response to jasmonic acid, *MYC2* is the core TF, and overexpression of *MYC2* can promote the production of phenolic acids in *S. miltiorrhiza*^42^. In this study, the differential expression of those genes in *S. miltiorrhiza* purple and green phenotypes was also significant. However, the complexity and integrity of biological processes, the complex regulatory network of plant anthocyanin metabolism, and various genetic and environmental factors affect the whole biosynthesis process.

Among the different metabolites, multiple carbohydrates were highly enriched, including d-glucose, l-rhamnose, d-galactose, d-xylose, and l-arabinose. Glycosylated forms of flavonoids and anthocyanins in flavonoids appear most often and are highly soluble in water. Therefore, hydrolysis may occur and other substances may form, resulting in a decrease in anthocyanin content. Moreover, the high expression of the glycosyltransferase 3AT could explain how glucose and flavonoids form anthocyanins through glycosylation, which eventually led to the difference in anthocyanin components between the two *S. miltiorrhiza* phenotypes. The DEGs and DAMs were more abundant in stems than in leaves, and expression in purple stems was relatively high. Flavonoids can be selectively transported from one organ to another in plants and from sites of synthesis to other parts^43^. Flavonoid distribution may be mediated by the action of MRP/ABC transporters rather than passive diffusion^44^. In this study, GST, ABC transporter, MATE, and SNARE genes were detected, which may have important roles in transporting anthocyanins to plant vacuoles. Therefore, some flavonoids and anthocyanins were likely transported to leaves by ABC transporters and plant hormone signal transduction, resulting in purple characters on the edges of some leaves. However, some anthocyanins might have been hydrolyzed during transport, which could also explain the accumulation of many of differential metabolites in the ABC transporter pathway and the variety of differential carbohydrate metabolites.

## Materials and methods

### Plant materials

*Salvia miltiorrhiza* seedlings were provided by Shaanxi Tasly Plant Medicine Co., Ltd. (Shangluo, China) and were identified by Prof. Yuan Chen (Gansu Agricultural University, Lanzhou City, Gansu Province). Seedlings were planted in the herbal garden of Gansu Agricultural University (Longitude: 103.700153, latitude: 36.090657). The collection of plant materials complied with relevant institutional, national, and international guidelines and legislation. Completely green stems (S1), purple stems (S2), completely green leaves (L1), and leaves with purple edges (L2) were collected in a full-blossom period, frozen with liquid nitrogen, and stored at − 80 °C. Samples from six individual plants were pooled as one biological replicate, The voucher specimens are deposited in the herbarium of medicinal plants of Gansu Agricultural University (Nos. GAUAB-SM-20210622-S1, GAUAB-SM-20210622-S2, GAUAB-SM-20210622-L1, and GAUAB-SM-20210622-L2). Three biological replicates were used in transcriptome and metabolome analyses conducted at the Shenzhen Genomics Institute (BGI) Co., Ltd.

### Transcriptomic sequencing

Because original sequence data obtained by sequencing could not be directly used for subsequent information analysis, the filtering software SOA Pnuke^45^ was used to filter out low-quality, joint pollution, and reads with high content of unknown base N (> 5%). The filtered data were considered “clean reads”^46^. The clean reads were assembled to obtain unigenes, and the unigenes were analyzed for function annotation, predicted coding area, and SSRs. The expression amount of each sample was calculated on the basis of all unigenes. The DEGs were screened according to *q*-value (adjusted P-value) ≤ 0.05. Based on the principle of negative binomial distribution, DEseq2 software was used for differential analysis^47^. The screening threshold was *q*-value ≤ 0.05 and log2 |(fold-change)|≥ 1^48^.

### Metabolomics analysis

Untargeted metabolomics analysis was performed using LC–MS/MS. The data obtained were processed by Compound Discoverer 3.1 (Thermo Fisher Scientific, USA)^49^. The metabolomics software package metaX and metabolomic information analysis processing were used for data preprocessing, statistical analysis, metabolite classification, and functional annotation^50^. This work used VIP values of the first two principal components in a multivariate Partial Least Squares Discriminant Analysis (PLS-DA) model^51^, combined with fold-change and *t*-test of univariate analysis to identify DAMs according to fold-change ≥ 1.2 or ≤ 0.83 and *P* < 0.05^52^.

### Combined analysis to reveal biosynthesis of flavonoids

Each metabolite may be regulated by multiple genes, or multiple metabolites may be regulated by one gene. The differential genes obtained by sequencing the transcriptome were targeted at the utilization site(http://www.plabipd.de/portal/mercator-sequence-annotation). The provided online annotation tool performed functional annotation classification, obtained a mapping file with similar structure, imported log2 fold-change data of DEGs, and then used MAPMAN software for joint analysis of transcriptional and metabolomic data^53^. Last, a working model was proposed to explain the internal mechanism of purple pigmentation in aboveground parts of *S. miltiorrhiza*, and a schematic diagram of flavonoid biosynthesis and regulation in *S. miltiorrhiza* was constructed.

### Reverse-transcription quantitative PCR expression analysis

Total RNA of *S. miltiorrhiza* stem and leaf was extracted according to a DP441 RNA Prep Prue Polysaccharide Polyphenol Plant Total RNA Extraction Kit (Tiangen Biotech, Beijing, China) and reverse-transcribed according to a PrimeScriptTM RT Reagent Kit with gDNA Eraser (Code No. RR047A; Takara Biotech, Beijing, China). The cDNA was used as the template to measure gene expression. The specific primers for genes involved in flavonoid biosynthesis and the *S. miltiorrhiza* actin gene (internal control) are listed in Supplementary Table S1. Reverse-transcription quantitative polymerase chain reaction (RT-qPCR) was conducted by a real-time PCR system (QuantStudio5) using TB Green® Premix ExT aq™ II (Code No. RR820A; Takara Biotech). Three biological replicates were used for qRT-PCR of each genes. The comparative CT method (2−ΔΔCTmethod) was used to quantify gene expression, firstly, for all test samples and calibration samples, CT values of actin gene are used to normalize CT values of target genes: ΔCT_(test)_ = CT_(target, test)_ − CT_(ref, test)_, ΔCT_(calibrator)_ = CT_(target, calibrator)_ − CT_(actin, calibrator)_. Secondly, the ΔCT value is the same as that of the test sample ΔCT value: ΔΔCT = ΔCT_(test)_ − ΔCT_(calibrator)_. Finally, the comparative CT method (2^−ΔΔCT^ method) was used to quantify gene expression. Statistical analysis was performed using Excel 2020 software (Microsoft Office, USA).

### Laser scanning confocal microscopy

Microstructure and distribution of flavonoids in aboveground parts of different *S. miltiorrhiza* phenotypes were evaluated by an LSCM method^54^. Flavonoids react with NA reagent (0.1% 2-aminoethyl diphenylborinate (w/v) in 80% methanol) and produce fluorescence^55^. The middle part of a stem or leave was cut into small pieces (width of 3 mm), which were then cut into 30-μm sections with a freezing microtome (CM1950; Leica, Germany). Sections required treatment to induce fluorescence. A drop of self-made phosphoric acid buffer solution (with the addition of 1% NaCl (w/v), pH 6.5) was added to the sections, followed by a drop of NA solution. After incubation for 5 min, sections were analyzed with LSCM (LSCM 800; Carl Zeiss, Germany).

## Conclusions

Contents of flavonoids and anthocyanins in the purple phenotype depended on the up-regulation of genes associated with biosynthesis of flavonoids and anthocyanins. The difference in color between purple and green phenotypes was the result of differential anthocyanin accumulation and differential gene expression. Those results and differences in morphological phenotypes suggested that regulation of structural genes in the flavonoid biosynthesis pathway promoted anthocyanin accumulation and contributed to the formation of the purple phenotype. The results clarify the important role of flavonoids in developmental processes and provide a basis for further understanding of organ coloring and the networks regulating flavonoid metabolism. Overall, the work revealed the dynamic changes in metabolites and the molecular regulation mechanisms underlying the color change in stem and leaf in *S. miltiorrhiza* and thus can also provide valuable theoretical support for further exploration of new medicines and health foods with flavonoids.

## Supplementary Information

Supplementary Information.

## Data Availability

The RNA-seq data sets for the transcriptome analysis were available at the Genome Sequence Archive in NCBI, under Biological Project ID: PRJNA796114. that are publicly accessible at https://www.ncbi.nlm.nih.gov/bioproject/PRJNA796114. The raw values obtained from the metabolomic data sets were available in Supplemental Data. If any data sets are unavailable through the links stated above, they can be obtained from the corresponding author on request.
