# Supplementary material for: Biosynthetic regulatory network of flavonoid metabolites in stems and leaves of Salvia miltiorrhiza
Source: Sci Rep. 2022 Oct 28;12:18212. doi: 10.1038/s41598-022-21517-5 (PMC9616839; doi:10.1038/s41598-022-21517-5)
Supplement: Supplementary file 1 — Supplementary Information. [file 41598_2022_21517_MOESM1_ESM.docx]

Supplementary Material

# Supplementary Figures and Tables

## Supplementary Figures


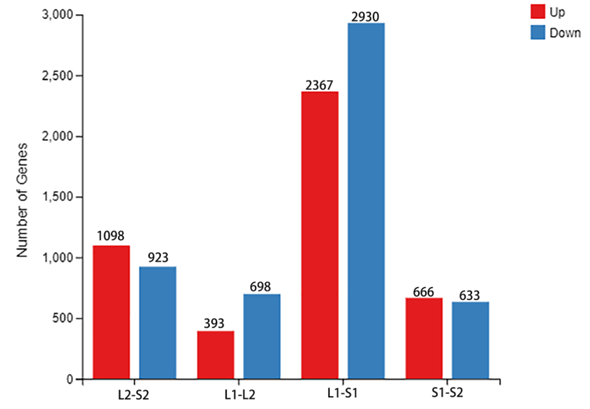


**Figure S1.** Quantity statistics of DEGs (differentially expressed genes); L1 (green leaf), L2 (leaves with purple edges), S1 (green stem), S2 (purple stem)


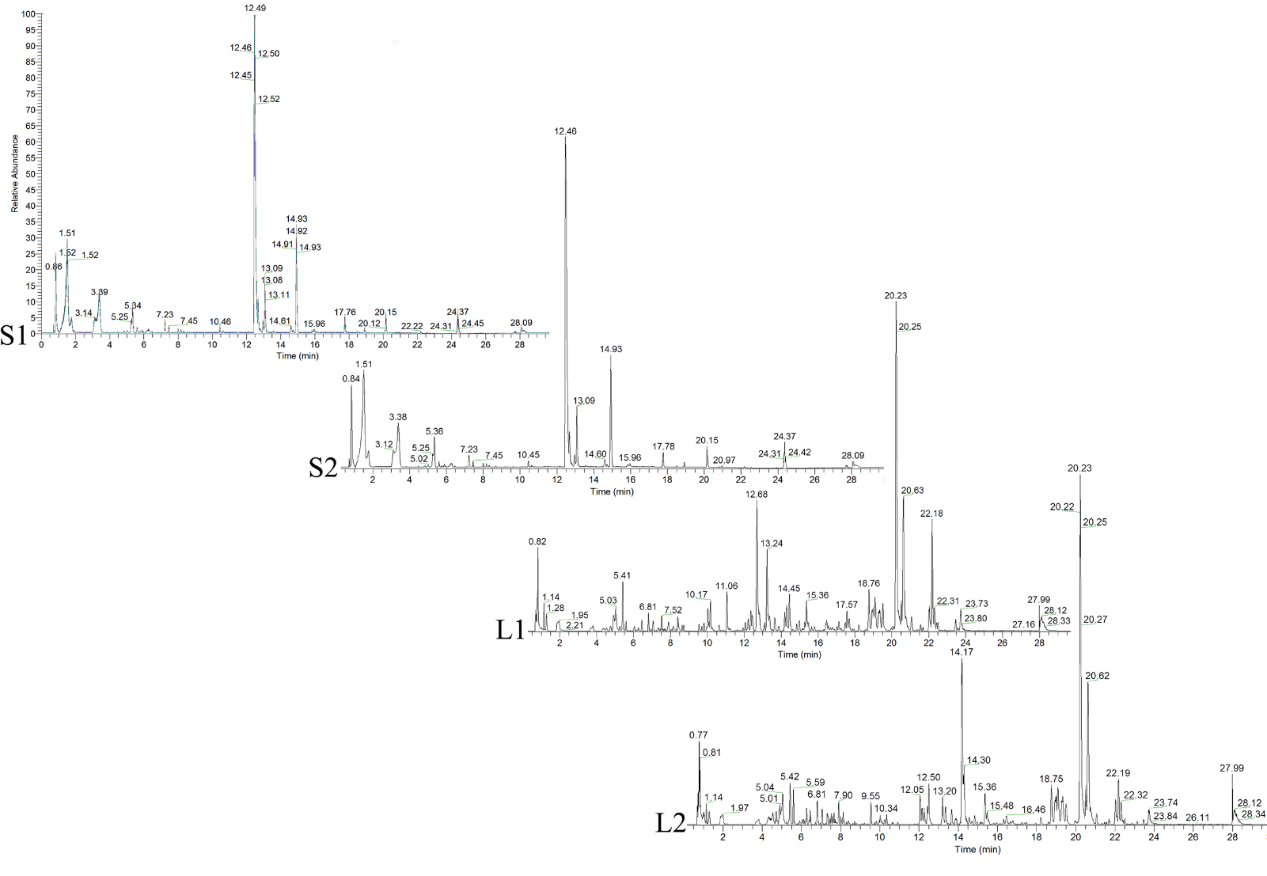


**Figure S2.** Total ion flow chromatogram of stems and leaves of *S. miltiorrhiza*


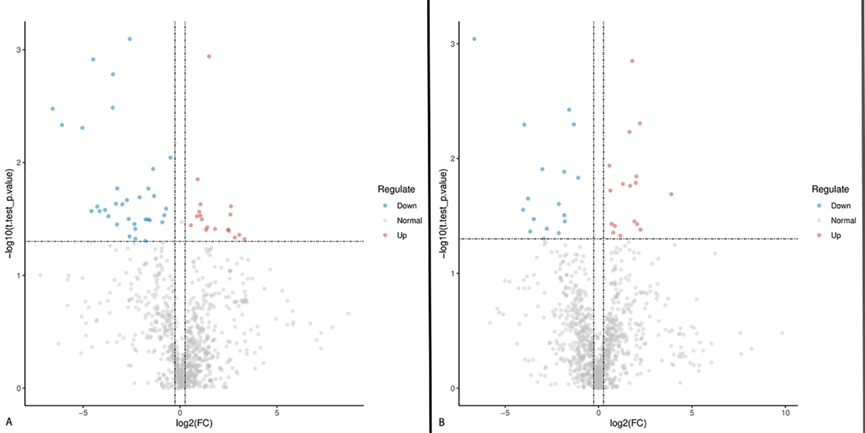


**Figure S3.** Volcanic map of DAMs (differentially accumulated metabolites)

**A:** Volcanic map of DAMs in S1-S2 **B:** Volcanic map of DAMs in L1-L2. L1 (green leaf), L2 (leaves with purple edges), S1 (green stem), S2 (purple stem)





**Figure S4.** Fluorescence intensity of flavonoids in stems and leaves of S. miltiorrhiza. S1 and S2 were three repeats of purple and green phenotypes, respectively

## Supplementary Tables

**Table S1:** Sequences of specific primers for qRT-PCR

| Primer name | Forward (5'-3') | | Reverse (5'-3') |
| --- | --- | --- | --- |
| CYP73A(C4H)  4CL  DFR  ANS  F3H  3AT  HCT  WRKY34  MYB34  MYB91  Actin | CCGGTGAAGATGTCGAACAC  GAAGAGTCCGGTGGTGGATA  GACGCCAAAGGCAGATACAT  TCCGCCGACTTCTTTCTCTA  CCACTCCGGCACGTATCTAT  CAATCTCCAACCCTCCTTCA  GCTCCGGCTGTGATAGGTAG  CTCCCACCGTCACTCTTGAT  TGGACCATCTGTTCCCTAGC  GGACTGCTGAGGAAGACAGG  TGCTGTGCTGAGGACGATAC | GTTTCGGCGACATCTTCCTC  CGAGCTTGGCATTAGGAAAC  CATCCTTCCACGAATCAGGT  CCAAGCACCCAGCTGATTAT  GCTCTCCTCCAGCTTCAATG  CGACCGAGGAAGTTTGAGTC  AAGAATCGACGATGGTGAGG  AGGTAGAGGCCATCTGCTCA  ACTTGCAGGTCTGCTGAGGT  CCTGCAAGTTTAGGGACAGC  CCATGAGCCTCCAAACCTAA | |

**Table S2:** Quality of the transcriptomes of aboveground in *S. miltiorrhiza*

| Sample | Total Clean Reads (M) | | Total Clean Bases (Gb) | Clean Q20 (%) | Clean Q30 (%) | Clean Reads Ratio (%) | Total Mapping (%) | | | Uniquely Mapping (%) | GC (%) |
| --- | --- | --- | --- | --- | --- | --- | --- | --- | --- | --- | --- |
| L1-1 | | 42.72 | 6.41 | 96.34 | 91.14 | 97.48 | 86.9 | | | 17.68 | 43.87 |
| L1-2 | | 42.54 | 6.38 | 96.12 | 90.64 | 97.07 | 87.2 | | | 14.44 | 43.93 |
| L1-3 | | 42.68 | 6.4 | 96.1 | 90.61 | 97.4 | 86.97 | | | 16.03 | 43.92 |
| L2-1 | | 42.67 | 6.4 | 96.82 | 91.97 | 97.37 | 86.24 | | | 15.96 | 44.03 |
| L2-2 | | 42.91 | 6.44 | 96.81 | 91.9 | 97.92 | 87.12 | | | 16.54 | 43.97 |
| L2-3 | | 42.85 | 6.43 | 96.87 | 92.05 | 97.78 | 86.81 | | | 16.66 | 44.1 |
| S1-1 | | 42.83 | 6.42 | 96.31 | 90.98 | 97.73 | | 86.45 | 20.78 | | 43.74 |
| S1-2 | | 42.51 | 6.38 | 96.38 | 91.25 | 97 | | 86.16 | 19.96 | | 44.05 |
| S1-3 | | 42.91 | 6.44 | 96.28 | 90.96 | 97.92 | | 86.16 | 18.72 | | 43.9 |
| S2-1 | | 42.9 | 6.43 | 96.39 | 91.23 | 97.89 | | 86.7 | 20.75 | | 43.8 |
| S2-2 | | 42.64 | 6.4 | 96.52 | 91.47 | 97.31 | | 86.76 | 19.76 | | 44.1 |
| S2-3 | | 42.83 | 6.42 | 96.37 | 91.14 | 97.73 | | 86.44 | 20.92 | | 44.1 |

**Table S3：**Candidate genes related to flavonoids biosynthesis in *S. miltiorrhiza* (The number of genes is in parentheses)

| Gene ID | Abbreviation | | Definition | [Length](javascript:;) | log2FC |
| --- | --- | --- | --- | --- | --- |
| Unigene7496_All | | PAL (7) | phenylalanine ammonia-lyase | 2582 | 4.30 |
| CL10126.Contig1_All | | 4CL (9) | 4-coumarate--CoA ligase | 986 | 5.21 |
| CL6660.Contig5_All | | CYP73A | trans-cinnamate 4-monooxygenase | 2376 | -2.12 |
| CL1935.Contig4_All | | DFR (3) | flavanone 4-reductase | 630 | -4.85 |
| Unigene1314_All | | ANS (2) | anthocyanidin synthase | 1286 | 4.82 |
| CL3911.Contig2_All | | FLS (4) | flavonol synthase | 2361 | -2.85 |
| CL10519.Contig2_All | | HCT | shikimate O-hydroxycinnamoyltransferase | 1105 | 4.01 |
| CL6678.Contig4_All | | 3AT | anthocyanidin 3-O-glucoside 6''-O-acyltransferase | 1627 | 3.35 |
| Unigene1080_All | | F3H | flavanone 3-hydroxylase | 1563 | 3.18 |

**Table S4**：Candidate genes related to flavonoids biosynthesis in *S. miltiorrhiza*

| Gene ID | Abbreviation | Gene length | P_value_ | FPKM | | | |
| --- | --- | --- | --- | --- | --- | --- | --- |
|  |  |  |  | **L1** | **L2** | **S1** | **S2** |
| CL1383.Contig5_All | bHLH (PIF4) | 1223 | 1.72 | 19.006 | 2.396 | - | - |
| CL2498.Contig5_All | bHLH (PIF3) | 1997 | 1.59 | 4.043 | 0 | 3.26 | 0.17 |
| CL7915.Contig12_All | bHLH (PIF4) | 2015 | 1.23×10^-7^ | 0.196 | 8.123 | 0.03 | 2.923 |
| CL2334.Contig2_All | bHLH6(MYC2)6 | 2276 | 0.97×10^-4^ | 26.62 | 12.10 | 99.02 | 9.503 |
| CL469.Contig3_All | WRKY13 | 1911 | 0.21×10^-3^ | 3.313 | 0.26 | 4.86 | 0.03 |
| CL782.Contig3_All | WRKY55 | 2074 | 2.02×10^-10^ | 3.056 | 0 | 1.18 | 0 |
| CL11393.Contig17_All | WRKY39 | 1413 | 0.53×10^-3^ | - | - | 10.176 | 0.05 |
| CL12543.Contig8_All | WRKY34 | 1544 | 0.21×10^-4^ | 64.5 | 2.88 | 124.86 | 1.48 |
| CL262.Contig1_All | WRKY24 | 1581 | 0.2×10^-5^ | 1.71 | 2.92 | 0 | 1.253 |
| CL6584.Contig5_All | WRKY48 | 1271 | 3.2×10^-7^ | 1.59 | 6.13 | 0 | 3.25 |
| CL4597.Contig11_All | MADS (5) | 997 | 0.0018 | 18.986 | 10.15 | 104.17 | 49.57 |
| Unigene10692_All | MADS(MEF2A)3 | 1311 | 1.2×10^-7^ | 14.99 | 6.88 | 0.41 | 5.75 |
| Unigene18002_All | bZiP43(2) | 792 | 0.62×10^-3^ | 0.73 | 24.97 | 0.28 | 25.136 |
| CL4727.Contig1_All | bZiP61 | 1540 | 1.6×10^-5^ | 5.426 | 0.98 | 0.39 | 0.14 |
| Unigene27410_All | bZiP (ATF2) | 1308 | 2.6×10^-7^ | 23.31 | 1.83 | 6.733 | 0.35 |
| Unigene17553_All | MYB46 | 1096 | 0.2×10^-4^ | - | - | 13.966 | 6.20 |
| CL9289.Contig3_All | MYB94 | 1534 | 1.88×10^-7^ | 0.193 | 6.09 | 0.23 | 6.79 |
| CL4418.Contig6_All | MYB(LHY) | 1972 | 0.8×10^-5^ | 4.52 | 0.18 | 3.33 | 0.233 |
| CL7621.Contig2_All | MYB92(2) | 1887 | 0.79×10^-4^ | - | - | 12.356 | 7.836 |
| CL3377.Contig2_All | MYB43(3) | 1350 | 0.26×10^-3^ | 2.593 | 24.746 | 2.43 | 29.533 |
| CL6988.Contig5_All | MYB91 | 1190 | 5.57×10^-11^ | 1.913 | 1.05 | 40.49 | 13.24 |
| CL6268.Contig3_All | MYB46 | 1087 | 1.14×10^-10^ | - | - | 26.83 | 11.43 |
| CL532.Contig14_All | MYB108 | 2975 | 3.26×10^-7^ | 0.09 | 3.13 | 0.01 | 6.093 |
| CL8731.Contig2_All | MYB39 | 1031 | 0.45×10^-4^ | 0.76 | 1.66 | 5.84 | 1.136 |
| Unigene21717_All | MYB24 | 1239 | 4.3×10^-7^ | 0.99 | 1.78 | 86.76 | 73.743 |
| Unigene26034_All | MYB3 | 1557 | 0.26×10^-3^ | 22.58 | 1.63 | 10.616 | 0.413 |
| Unigene21822_All | MYB5 | 912 | 0.16×10^-3^ | 5.136 | 0.473 | 11.13 | 1.41 |
| CL6988.Contig4_All | MYB91(2) | 1524 | 0.16×10^-4^ | 0.63 | 0.336 | 6.82 | 3.07 |
| CL7905.Contig2_All | MYB37 | 1007 | 2.85 | - | - | 11.75 | 6.01 |
| CL10400.Contig2_All | MYB4 | 1699 | 0.37×10^-5^ | - | - | 14.696 | 5.58 |
| CL4639.Contig4_All | MYB101 | 1434 | 5.05×10^-8^ | - | - | 47.926 | 51.57 |

**Table S5:** List of differential metabolites in S1-S2

| **ID** | **P** | **Regulate** | | **Name** |
| --- | --- | --- | --- | --- |
| **1.212_283.09167** | 1.498 | Up | Guanosine | |
| **1.35_285.08495** | 1.455 | Down | Luotonin A | |
| **1.962_330.07165** | 1.853 | Up | Aflatoxin G2 | |
| **10.159_507.10273** | 1.412 | Up | 1,4:3,6-Dianhydro-2-deoxy-5-O-[(4-methoxyphenyl)carbamoyl]-  2-[(8-quinolinylsulfonyl)amino]-D-glucitol | |
| **10.651_368.19646** | 1.534 | Down | 1-methyl-2-{(3s)-1-[4-(2-pyridinyl)benzyl]-3-pyrrolidinyl}-1h-benzimidazole | |
| **10.73_294.21945** | 2.487 | Down | 9-Oxo-ODE | |
| **12.451_417.21519** | 1.472 | Down | Protostemonine | |
| **12.954_320.23509** | 1.569 | Down | (5ξ,9ξ,16ξ)-17-Hydroxykauran-19-oic acid | |
| **13.925_357.15688** | 1.630 | Up | Deschloro W-19 | |
| **14.169_190.09972** | 2.308 | Down | Ligustilide | |
| **15.939_310.15686** | 1.570 | Down | Estriol | |
| **16.692_321.26673** | 1.425 | Up | α-Linolenoyl ethanolamide | |
| **17.104_297.26663** | 1.592 | Down | 2-Aminooctadec-4-yne-1,3-diol | |
| **17.839_323.28236** | 1.404 | Up | Linoleoyl ethanolamide | |
| **17.994_304.24025** | 1.345 | Down | Arachidonic acid | |
| **18.818_319.25050** | 2.941 | Up | 17α-Methyl-androstan-3-hydroxyimine-17β-ol | |
| **18.944_306.25548** | 1.631 | Down | Linolenic acid ethyl ester | |
| **19.411_310.15649** | 1.611 | Down | (3E,12E)-3,12-Dimethyl-8-methylene-6,18-dioxatricyclo[14.2.1.05,9]  nonadeca-3,12,16(19)-triene-7,17-dione | |
| **19.479_361.20399** | 2.915 | Down | Octocrylene | |
| **19.89_336.15984** | 1.445 | Up | 6-Benzyl-2-(tert-butyl)-6,7-dihydro-4H-pyrazolo[1,5-a]pyrrolo[3,4-d]pyrimidine-5,8-dione | |
| **2.977_322.06642** | 3.096 | Down | N'2-{2-[(4-Chlorophenyl)thio]ethanimidoyl}-1-methyl-1H-pyrrole-2-carbohydrazide | |
| **20.874_496.35297** | 1.667 | Down | Diundecyl phthalate | |
| **3.868_297.08951** | 1.524 | Up | 5'-S-Methyl-5'-thioadenosine | |
| **3.951_368.05095** | 1.497 | Down | O1-{[5-(3-Thienyl)-1,2,4-oxadiazol-3-yl]methyl}-4-(trifluoromethyl)  benzene-1-carbohydroximamide | |
| **4.319_146.03703** | 1.408 | Up | Coumarin | |
| **4.36_164.04759** | 1.541 | Up | 4-Coumaric acid | |
| **4.363_274.04128** | 2.333 | Down | 2-Phenylbenzimidazole-5-sulfonic acid | |
| **4.969_498.17188** | 1.770 | Down | 1-[(1r,9s)-11-(4-fluorobenzoyl)-6-oxo-7,11-diazatricyclo[7.3.1.02,7]  trideca-2,4-dien-5-yl]-3-(2-methoxyphenyl)urea | |
| **5.013_434.11917** | 2.782 | Down | N-{(3s,5s)-5-[3-(4-chlorophenyl)-1,2,4-oxadiazol-5-yl]-  1-methyl-3-pyrrolidinyl}-2-phenoxyacetamide | |
| **5.084_408.13969** | 1.413 | Down | Tinnevellin glucoside | |
| **5.084_424.11375** | 1.324 | Down | Nobiletin | |
| **5.464_262.12056** | 2.044 | Down | (3aS,10aR,10bR)-6,10a-Dimethyl-3-methylene-3,3a,4,5,7,8,10a,10b  -octahydrofuro[3',2':6,7]cyclohepta[1,2-b]pyran-2,9-dione | |
| **5.555_224.06876** | 1.306 | Down | Sinapic acid | |
| **5.715_478.14780** | 1.944 | Down | Calceolarioside B | |
| **5.719_264.06343** | 1.703 | Down | Sulfamerazine | |
| **6.144_475.29969** | 1.321 | Up | PEG n10 | |
| **6.226_358.06880** | 1.563 | Up | Dichotomitin | |
| **6.64_563.35178** | 1.360 | Up | PEG n12 | |
| **6.718_458.11919** | 1.496 | Down | Phlorizin | |
| **6.719_453.16377** | 1.490 | Down | (2S,3R)-3,5-Dihydroxy-2-(4-hydroxyphenyl)-3,4-dihydro-2H  -chromen-7-yl β-D-glucopyranoside | |
| **6.856_607.37863** | 1.337 | Up | PEG n13 | |
| **7.056_651.40448** | 1.612 | Up | PEG n14 | |
| **7.665_340.09464** | 1.529 | Up | (6aR,12aR)-12a-Hydroxy-2,3,9-trimethoxy-6a,12a-dihydrochromeno  [3,4-b]chromen-12(6H)-one | |
| **7.793_530.17717** | 1.692 | Down | 4-(2-{[1-amino-3-(4-methoxyphenyl)-1-oxo-2-propanyl]carbamoyl}-  4-{[(methylsulfanyl)acetyl]amino}-1-piperidinyl)-4-oxobutanoic acid | |
| **7.9_356.12591** | 1.395 | Up | [(3S)-3-(5-Benzyl-1,3,4-oxadiazol-2-yl)-1-pyrrolidinyl](4-pyridinyl)methanone | |
| **8.427_142.09964** | 1.771 | Down | Cyclohexaneacetic acid | |
| **8.428_510.26586** | 1.524 | Down | 6-Hydroxy-1-(hydroxymethyl)-5-{2-[2-(hydroxymethyl)-1-pyrrolidinyl]  -2-oxoethyl}-1,4a-dimethyldecahydro-2-naphthalenyl phenylcarbamate | |
| **8.43_486.18946** | 2.477 | Down | Rutaevin | |
| **8.43_526.23974** | 1.451 | Down | N-{[(2r,4s,5s)-5-{[4-(4-fluorophenyl)-1-piperazinyl]methyl}-1-  azabicyclo[2.2.2]oct-2-yl]methyl}-4-(trifluoromethyl)benzamide | |
| **8.431_220.18298** | 1.635 | Down | (-)-Caryophyllene oxide | |
| **8.432_521.28424** | 1.580 | Down | Taurodeoxycholic acid | |
| **9.742_185.21470** | 1.500 | Down | Tributylamine | |
| **15.939_310.15686** | 1.570 | Down | Estriol | |

**Table S6:** List of differential metabolites in L1-L2

| **ID** | **P** | **Regulate** | **Name** |
| --- | --- | --- | --- |
| **0.824_542.12578** | 1.722 | Up | Rhusflavanone |
| **0.825_161.10545** | 1.780 | Up | Stachydrine |
| **0.828_302.04036** | 1.763 | Up | Herbacetin |
| **0.828_348.08215** | 1.939 | Up | 2--(tert-Butyl)-8-[(3,4-dichlorobenzyl)oxy]imidazo[1,2-a]pyridine |
| **1.144_138.04314** | 1.789 | Up | Urocanic acid |
| **10.702_529.28942** | 1.554 | Down | 3-O-β-D-Glucopyranosylandrographolide |
| **11.133_316.09464** | 1.652 | Down | Byakangelicol |
| **16.25_299.28238** | 2.295 | Down | Palmitoyl ethanolamide |
| **16.634_390.25593** | 1.355 | Up | 2,2-Methylenebis(4-ethyl-6-tert-butylphenol) |
| **16.686_321.26675** | 1.431 | Up | α-Linolenoyl ethanolamide |
| **18.753_328.23985** | 1.473 | Down | Docosahexaenoic acid |
| **19.24_325.29773** | 2.307 | Up | Oleoyl ethanolamide |
| **19.354_327.31355** | 1.366 | Down | Stearoyl ethanolamide |
| **19.455_178.06318** | 1.381 | Up | 4-Methoxycinnamic acid |
| **19.825_454.34482** | 1.389 | Down | Liquidambaric acid |
| **2.221_237.06391** | 2.426 | Down | Kinetin |
| **20.036_454.34484** | 1.885 | Down | Oleanonic acid |
| **3.87_204.09019** | 1.453 | Up | DL-Tryptophan |
| **3.872_187.06364** | 1.846 | Up | Indole-3-acrylic acid |
| **3.997_300.11849** | 1.329 | Up | 7-(4-Methoxybenzyl)-1,3-dimethyl-3,7-dihydro-1H-purine-2,6-dione |
| **4.027_252.11113** | 1.690 | Up | Tyrosylalanine |
| **4.362_274.04128** | 1.303 | Down | 2-Phenylbenzimidazole-5-sulfonic acid |
| **4.539_513.20772** | 1.414 | Up | 4-Fluoro-N-[7-hydroxy-8-(hydroxymethyl)-4a,8-dimethyl-4-{2-[methyl(2-propyn-1-yl)amino]-  2-oxoethyl}-4,4a,5,6,7,8,8a,9-octahydronaphtho[2,3-d][1,3]thiazol-2-yl]benzamide |
| **4.566_236.11627** | 1.429 | Up | 3-Morpholino-4-tetrahydro-1H-pyrrol-1-ylcyclobut-3-ene-1,2-dione |
| **5.082_424.11366** | 1.453 | Down | Nobiletin |
| **5.085_224.10510** | 1.351 | Down | Senkyunolide H |
| **5.671_202.12087** | 1.833 | Down | Sebacic acid |
| **6.971_358.12398** | 2.232 | Up | 1-(3-{[(5-Acetyl-2-methoxybenzyl)thio]methyl}-4-methoxyphenyl)ethan-1-one |
| **7.333_145.05300** | 2.850 | Up | 4-Indolecarbaldehyde |
| **7.015_224.10509** | 1.507 | Down | Pogostone |
| **7.409_418.18408** | 1.604 | Down | (1r,9s)-11-(2-pyrazinylcarbonyl)-3-[4-(trifluoromethoxy)phenyl]-7,11-diazatricyclo  [7.3.1.02,7]trideca-2,4-dien-6-one |
| **7.529_268.13112** | 2.298 | Down | 2-(1-Ethyl-3-methyl-1H-pyrazol-5-yl)-5-(4-methylphenyl)-1,3,4-oxadiazole |
| **8.419_486.18939** | 3.042 | Down | Rutaevin |
| **9.63_222.08945** | 1.908 | Down | Monobutyl phthalate |
